# Cutaneous Anthrax, West Bengal, India, 2007

**DOI:** 10.3201/eid1503.080972

**Published:** 2009-03

**Authors:** Tapas K. Ray, Yvan J. Hutin, Manoj V. Murhekar

**Affiliations:** National Institute of Epidemiology, Chennai, India

**Keywords:** anthrax, outbreak, India, letter

**To the Editor:** In most of India, anthrax is not common, probably because a large proportion of the population is Hindu and does not eat beef. However, sporadic cases and outbreaks have been reported (*1**–**6*).

On June 8, 2007, a healthcare facility reported 12 cases of cutaneous anthrax in the Muslim village of Sarkarpara (population 361). On August 4, 2007, another facility 50 km away reported 8 cases from the Muslim village of Charbinpara (population 835). These 2 outbreaks, both in Murshidabad district, West Bengal, were associated with the slaughtering of 4 cows. We investigated each outbreak to confirm diagnosis, estimate magnitude (incidence and severity), and identify risk factors. We conducted house-to-house searches to identify case-patients and collected smears from skin lesions.

From Sarkarpara, we identified 45 cases of cutaneous anthrax and 2 deaths (attack rate 12%, case-fatality rate 4%); from Charbinpara, we identified 44 cases and no deaths (attack rate 5%). In Sarkarpara, villagers had slaughtered a cow on June 2, 2007. The outbreak started on June 3, peaked on June 6 (1 cluster), and ended on June 10. In Charbinpara, villagers had slaughtered 3 cattle, 1 each day, on July 16, July 23, and August 1. The first case occurred on July 17 and was followed by 3 peaks (3 clusters) (Figure). In each village, attack rates were highest among persons 15–44 years of age. Microscopic examination at the district public health laboratory showed gram-positive, spore-bearing bacilli that were characteristic of *Bacillus anthracis* on 7 of 20 smears (5/10 from Sarkarpara and 2/10 from Charbinpara).

**Figure:**
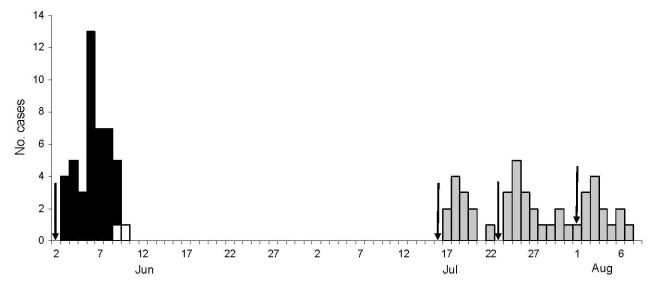
Cases of cutaneous anthrax, Mushidabad district, West Bengal, India, 2007. Dates indicate onset of skin lesion. Arrows indicate dates cattle were slaughtered. Black bars, cases in Sarkarpara village; gray bars, cases in Charbinpara village; white bars, deaths.

To test the hypothesis that exposure to meat of a slaughtered cow was associated with illness, we conducted a retrospective cohort study among families who had handled or eaten beef from cows slaughtered during the week before the outbreak. Through interviews, we collected information about possible exposures, including slaughtering, handling meat or skin, and eating beef.

In Sarkarpara, we enrolled 296 persons from 59 families in the cohort study. Persons who had slaughtered cows and handled meat and skins had a significantly higher risk for illness than those who had not. In Sarkarpara, risk associated with slaughtering cattle was 9.1 (95% confidence interval [CI] 6.0–13.7) and with handling meat 2.6 (95% CI 1.5–4.4) (Appendix Table). Slaughtering cows or handling meat accounted for the largest proportion of cases; 8% and 33% of the population was engaged in these practices, respectively (population-attributable fraction [PAF] 39% [95% CI 37.0–41.2] and 34% [95% CI 18.5–42.9], respectively). PAF associated with handling skins was 2% (95% CI 1.8–2.0).

In Charbinpara, we included 687 persons from 118 families in the cohort study. Slaughtering cattle and distributing beef were strongly associated with illness (Appendix Table). Slaughtering cows and handling meat were common practices and accounted for the largest proportion of cases (PAF 47% [95% CI 46.0–48.0] and 19% [95% CI 17.5–19.4], respectively). In Charbinpara, risk associated with slaughtering was 19.0 (95% CI 11.0–30.0) and with distributing was 11.0 (95% CI 6.8–19.0) (Appendix Table). Of the persons who ate beef, anthrax developed in 17% in Sarkarpara and 7% in Charbinpara. However, when we restricted the analysis to those who did not handle meat or skin, eating beef was not associated with illness. No person whose sole exposure was eating beef became ill. Persons who slaughtered cattle were not in the butchering profession; they did not wear gloves or other protective equipment. Their helpers distributed the beef in the village without any protection. Persons involved in skin trading carried the skins to nearby villages to sell. Women in the villages boiled the beef for 30 minutes before serving.

In Sarkarpara, healthcare workers knew the symptoms suggestive of anthrax and that this disease needed to be reported. As a result, this outbreak was reported early. In Charbinpara, healthcare workers knew nothing about the disease and did not report it. As a result, reporting was delayed until the third cluster. Late reporting prevented effective public health action. Because the source of infection in the 2 villages differed (different cattle), we were unable to formally establish a causal link between these 4 clusters.

Because the anthrax outbreak in Murshidabad was associated with slaughtering of ill cows and handling raw meat without taking any protective measures, we propose several recommendations. First, healthcare workers in anthrax-endemic areas need to be educated about promptly recognizing and reporting the disease. Second, persons in the community must be educated about using personal protective equipment during slaughtering of animals and handling of meat and skins. Community education should focus on those at risk, including Muslim communities who eat beef. Because anthrax occurs in only a few districts, India does not have a nationally organized control program (*7*). However, a focal prevention plan based on these recommendations would ultimately help reduce illness and death in anthrax-endemic districts.

## Supplementary Material

Appendix Table Risk for cutaneous anthrax according to selected exposures, 2 villages, Murshidabad district, West Bengal, India, 2007*
