# Supplementary material for: Cutaneous Anthrax, West Bengal, India, 2007
Source: Emerg Infect Dis. 2009 Mar;15(3):497–9. doi: 10.3201/eid1503.080972 (PMC2666291; doi:10.3201/eid1503.080972)
Supplement: Appendix Table — Risk for cutaneous anthrax according to selected exposures, 2 villages, Murshidabad district, West Bengal, India, 2007* [file 08-0972_appT-s1.pdf]

Appendix Table. Risk for cutaneous anthrax according to selected exposures, 2 villages, Murshidabad district, West Bengal, India, 2007*

	Sarkarpara village						Charbinpara village					
	Risk among exposed		Risk among unexposed		Association		Risk among exposed		Risk among unexposed		Association	
Characteristics of persons	Total no.	No. (%) cases	Total no.	No. (%) cases	RR	95% CI	Total no.	No. (%) cases	Total no.	No. (%) cases	RR	95% CI
Age >median†	163	30 (18)	133	15 (11)	1.6	0.9–2.9	351	26 (7)	336	18 (5)	1.4	0.8–2.5
Female sex	142	24 (17)	154	21 (14)	1.2	0.7–2.1	189	21 (11)	498	23 (5)	2.4	1.4–4.2
Slaughtered cattle‡	24	20 (83)	272	25 (9)	9.1	6.0–13.7	35	22 (63)	652	22 (3)	19	11–30
Distributed beef‡	–	–	–	–	–	–	15	9 (60)	672	35 (5)	11	6.8–19
Cleaned beef‡	–	–	–	–	–	–	174	12 (7)	513	32 (6)	1.1	0.6–2.1
Handled raw beef‡	97	25 (20)	199	26 (10)	2.6	1.5–4.4	–	–	–	–	–	–
Carried animal skins‡	1	1 (100)	295	44 (15)	6.7	5.1–8.8	2	1 (50)	685	43 (6)	8.0	1.9–32.8
Ate beef‡	260	45 (17)	36	0	–	–	654	44 (7)	33	0	–	–
Ate but did not handle beef	142	0	33	0	0	0	445	0	16	0	0	0

*RR, relative risk; CI, confidence interval; –, data not collected.
†Median age 20 years.
‡Involved sick cattle.
